# Supplementary material for: Patterns of Intron Gain and Loss in Fungi
Source: PLoS Biol. 2004 Nov 30;2(12):e422. doi: 10.1371/journal.pbio.0020422 (PMC532390; doi:10.1371/journal.pbio.0020422)
Supplement: Table S1 — Also available at http://genes.mit.edu/NielsenEtAl/. (4.3 MB ZIP). [file pbio.0020422.st001.zip › NielsenEtAl/html/441.html]

AN2332.1.NCU00959.1.MG00167.1.FG05610.1


```
 CLUSTAL W (1.82) Multiple Sequence Alignments - Introns Inserted


Sequence 1: MG00167.1	274 aa
Sequence 2: FG05610.1	260 aa
Sequence 3: NCU00959.1	282 aa
Sequence 4: AN2332.1	273 aa
Alignment Length: 286 aa
Number Identitical Residues: 163 aa
Alignment Score (without introns) 7972


MG00167.1 	MASLRSTTRLFASARPSAALRKPAMVSVRSMASVGE--~---------SESKMKTFQIYR
NCU00959.1	MAALRSSSARVFAAASRPAFR--PVVAARGMANLADGA~TQSQQASSEQSPKLKTFQIYR
FG05610.1 	MAALRSSSRLVGAFSRTAAIR-PGTVFTRSMASVSEPA~K-------EPESKLKSFQIYR
AN2332.1  	MASLRTTSRLVASSRS--LFR--PATFARSYATVESAQ0--------EPAPQMKKFQVYR
          	**:**:::  . :      :*    . .*. *.: .           .  .::*.**:**

MG00167.1 	WDPSQPSEKPRMQTYTLDLNKTGPMVLDALVRIKNELDPTLTFRRSCR~EGICGSCAMNI
NCU00959.1	WNPDTPTEKPRMQSYTLDLNKTGPMVLDALVRIKNELDPTLTFRRSCR~EGICGSCAMNI
FG05610.1 	WNPDTPSEKPRLQTYTLDLNKTGPMILDALIRIKNELDPTLTFRRSCQ1Y----------
AN2332.1  	WNPDKPSEKPKMQTYELDLKKTGPMMLDALIRIKNEIDPTLTFRRSCR~EGICGSCAMNI
          	*:*. *:***::*:* ***:*****:****:*****:**********:  . ..:.: . 

MG00167.1 	NGTNTLACLC1RIPTEASSEVKIYPLPHTYVVKDLVPDLTHFYKQYRSIKPYLQRDEPAP
NCU00959.1	NGTNTLACLC1RIPADNSAEMKIYPLPHTYVVKDLVPDLTLFYKQYKSIKPYLQRDTPSP
FG05610.1 	--------PG~RIPTEAASDVKIYPLPHTYVVKDLVPDLTHFYKQYKSIKPYLQRDTPAE
AN2332.1  	DGVNTLACLC1RIPTDTTKESRIYPLPHTYVVKDLVPDLTQIYKQYKSIKPYLQRDTKTE
          	.. .: :.   ***:: : : :****************** :****:*********  : 

MG00167.1 	D0GKEYRQSKEDRRKLDGLYECILCACCSTSCPSYWWNSEEYLGPAILLQSYRWLADSRD
NCU00959.1	D0GKEYRQSKADRKKLDGLYECILCACCSTSCPSYWWNSEEYLGPAILLQSYRWLADSRD
FG05610.1 	D0GREYRQTKEDRRKLDGLYECILCACCSTSCPSYWWNSEEYLGPAILLQSYRWLADSRD
AN2332.1  	D0GLENRQSPEDRKKLDGLYECILCFCCSTSCPSYWWNSEEYLGPAILLQSYRWLADSRD
          	* * * **:  **:*********** **********************************

MG00167.1 	QRKVERRLALDNSMSLYRCHTILNCTRTCPKGLNPAKAIAEIKKEMAFV-
NCU00959.1	ERTAERKDALNNSMSLYRCHTILNCTRTCPKGLNPGLAIANIKKELAF--
FG05610.1 	QRTAERKQNLENSMSLYRCHTILNCTRACPKGLNPGKAIAEIKKQMAFGN
AN2332.1  	QKTAERKHAIDNSMSVYRCHTILNCTRTCPKGLNPARAISEIKKMLAAH-
          	::..**:  ::****:***********:*******. **::*** :*
```
